# Retraction: Oxidation of Helix-3 Methionines Precedes the Formation of PK Resistant PrP^Sc^

**DOI:** 10.1371/journal.ppat.1012775

**Published:** 2024-12-11

**Authors:** The PLOS Pathogens Editors

Following the publication and subsequent Correction of this article [1, 2], additional concerns were raised regarding results presented in Figs 2 and 3. Specifically,

- Lanes 1–3 of the Fig 2C 6H4 MO panel appear similar to lanes 1–3 of the Fig 3A mAb IPC1 Mouse Brain panel.
- In the Fig 2C RVC panel, lane 11 appears similar to lanes 4–6 when horizontally stretched.

The authors provided uncropped blots for Figs 2, 3, 4, and 5BC in the Supporting Information files available with the previous Correction of this article [2]. Editorial review concluded that the data provided in [2] do not resolve the additional concerns raised with this article. The underlying mAb IPC1 mouse brain blot for Fig 3A provided with [2] appears to show the same data as the underlying blot provided for the Fig 2C 6H4 panel despite representing different experimental results. Regarding the Fig 2C RVC panel, the underlying data provided for this panel do not fully match the published panel, and underlying data provided for the Fig 2C R1 panel appear to partially match the published RVC panel. The data provided for these panels lend further support for the editorial concerns about the integrity of the data reported for the RVC hamster results and the RVC human E200K results.

The issues were not resolved in follow-up discussions.

Therefore, the *PLOS Pathogens* Editors retract this article due to concerns about the reliability and integrity of the published results.

RuthG did not agree with the retraction. TC, KF, RonenG, SL, AF, JM, and MG either did not respond directly or could not be reached.

The following additional issues were noted in our editorial assessment:

- The originally published Fig 4 mAb 6H4 Sc(RML) Mo panel of [1] appears similar to the Fig 6 Anti PrP pAb RTC Mouse RML panel of [3, retracted in 4]. The Fig 4 mAb 6H4 Sc(RML) Mo panel was corrected in [2] but the underlying data for this panel (provided in the S1 File available in [2]) confirm the originally published panel was inappropriately manipulated.
- Multiple blots provided with [2] as underlying data for Fig 4 do not appear to match the published panels; these may represent replicate data.
- There appears to be a labelling discrepancy between Fig 3B and its legend, which refer to IPC1, IPC2, RVC (Legend) vs. 6H4, IPC2, RVC (Figure).
